# Fetal face shape analysis from prenatal 3D ultrasound images

**DOI:** 10.1038/s41598-023-50386-9

**Published:** 2024-02-22

**Authors:** Raphael Sivera, Anna E. Clark, Andrea Dall’Asta, Tullio Ghi, Silvia Schievano, Christoph C. Lees

**Affiliations:** 1 Institute of Cardiovascular Science, University College London, London, UK; 2 Institute of Reproductive and Development Biology, Department of Metabolism, Digestion and Reproduction, Imperial College London, London, UK; 3 Department of Medicine and Surgery, University of Parma, Parma, Italy

**Keywords:** Translational research, Computational science, Intrauterine growth

## Abstract

3D ultrasound imaging of fetal faces has been predominantly confined to qualitative assessment. Many genetic conditions evade diagnosis and identification could assist with parental counselling, pregnancy management and neonatal care planning. We describe a methodology to build a shape model of the third trimester fetal face from 3D ultrasound and show how it can objectively describe morphological features and gestational-age related changes of normal fetal faces. 135 fetal face 3D ultrasound volumes (117 appropriately grown, 18 growth-restricted) of 24-34 weeks gestation were included. A 3D surface model of each face was obtained using a semi-automatic segmentation workflow. Size normalisation and rescaling was performed using a growth model giving the average size at every gestation. The model demonstrated a similar growth rate to standard head circumference reference charts. A landmark-free morphometry model was estimated to characterize shape differences using non-linear deformations of an idealized template face. Advancing gestation is associated with widening/fullness of the cheeks, contraction of the chin and deepening of the eyes. Fetal growth restriction is associated with a smaller average facial size but no morphological differences. This model may eventually be used as a reference to assist in the prenatal diagnosis of congenital anomalies with characteristic facial dysmorphisms.

## Introduction

Prenatal diagnosis of congenital anomalies is important for patient care and to facilitate parents’ counselling, planning of delivery, and postnatal treatment^1^. However, prenatal detection of fetal dysmorphisms is challenging because of the wide range of morphological features involved, the limitation of prenatal imaging^2^, and because the phenotypical descriptions of abnormalities seen on 2D ultrasounds are not always linked to easily identifiable genetic mutations. In this context, the analysis of fetal facial morphology in 3D can provide relevant information and serve as a pre-screening tool, facilitating the early detection of developmental disorders and genetic syndromes^3–7^.

Ultrasound imaging is routinely performed for fetal assessment, as it is non-invasive and nowadays widely available in middle and higher income countries. Three-dimensional ultrasound (3D US) was introduced clinically in the late 1990s and studies have explored the added value of 3D US for the assessment of fetal abnormalities^8–11^. Technical improvements have increased the quality of the images and their visualisation^12^. Yet, the use of 3D images has not truly permeated diagnostic fetal medicine and the analysis of these has almost exclusively been confined to qualitative assessments, vulnerable to misinterpretation. Noisy images, possible occlusion of the face, and overall acquisition variability (contrast, fetal position, maternal body habitus, amniotic fluid volume and operator handling) make the analysis of the images difficult. In particular, automatic morphometric approaches need to address several technical challenges to be able to assist the clinical analysis of the fetal face from 3D US.

Segmentation algorithms developed for other image modalities may not be suitable for ultrasound imaging because of the specific intensity, noise and image configuration of ultrasound. Intensity models for classification between fetal tissue and amniotic fluid in prenatal ultrasounds have been designed^13^, and specific approaches that process the images from different view-points to correct for orientation and position changes have been proposed^14^. However, these methods are not able to fully reconstruct the fetal face when the fetus is laid on the mother’s tissue. Similarly, deep learning approaches have been proposed^15,16^ but the task difficulty and the limited existing ground-truth data that could be leveraged to build and validate a new model limit the quality of the results, and the reconstructions are often rough or incomplete. The method proposed by Alomar et al.^17^ demonstrates promising results, is based on a model developed for the faces of newborn babies and requires the manual placement of a large number of landmarks. The reconstructions therefore approximate more closely to a newborn rather than fetal fetal face shape, particularly for earlier gestational ages. Many works still rely on manual slice-by-slice segmentation^18^.

Morphable face models have shown their potential to help diagnose congenital abnormalities from 3D scans^19,20^, including in newborns^21^. Prenatal diagnosis has been however more challenging. Deep-learning-based approaches have been recently developed for the recognition of facial expression associated with fetal brain development stages^22^ or for the prenatal screening of genetic conditions^23^ from 3D images. They are however limited by the interpretability of their features and their generalizability. In 2017, our group demonstrated the potential of shape modelling of the fetal face to assist pre-natal diagnosis and help characterise fetal face morphology from 3D ultrasound in a feasibility study^24^ with landmark-free morphometry^25^. However the study required time-consuming manual segmentation of the fetal face which limited the scope of the study and would prevent its clinical use. As such we latter developed a semi-automatic processing pipeline to segment 3D US fetal faces volumes^26^ using an atlas-based segmentation algorithm^27^: a set of manually labelled images, the atlas, is non-linearly registered on the image to segment, then, a probabilistic labelling map is obtained by merging a selected subset of candidate segmentations^28^.

This study extends and builds on the methodology proposed in our previous work in two ways. First, a more efficient semi-automatic segmentation workflow to allow the 3D reconstruction of a larger number of cases with a wider spectrum of clinical conditions is presented. Second, the statistical models have been extended to provide 3D description of the normal fetal face in the third trimester including the morphological change with advancing gestational age and the normal shape variability visible in a control population. The aim is to provide a reference range against which individual clinical conditions can be compared and outliers can be identified.

## Results

### Data and participants

235 participants (238 fetuses) were recruited to the study. Exclusion criteria were based on the absence of suitable 3D US volumes between the 24 and 34 week of gestation, or incomplete follow-up data. We focused on patients without any diagnosed facial dysmorphism with a final data set consisting of 135 fetuses, 117 appropriate grown (AGA) normal fetuses and 18 fetal growth restricted (FGR) normal fetuses. Participants were 18 to 52 years old (mean 32.9 years old), had a body mass index between 17 and 41 kg/m^2^ (mean 24.7 kg/m^2^), with fetuses of gestational ages (GA) from 24+0 weeks to 34+0 weeks. Mean gestational age is 28+4 weeks (standard deviation 2+6 weeks) for normal AGA, and 29+1 weeks (standard deviation 2+6 weeks) for normal FGR.

### Patient population and image acquisition

3D US volumes were obtained in the highest resolution acquisition mode on the ultrasound machine. The volumes were collected by fetal medicine staff experienced in performing ultrasound and no additional training was required. We estimate that an additional 10-15 minutes was required for each clinical appointment to collect the 3D volumes. It was possible to collect 3D facial volumes for 84% of recruited participants (n=204), for the remaining 16% fetal position precluded 3D volume acquisition. 3D US volume was collected during the first clinical visit in 92% of cases, the remaining 8% required between 2-4 clinical appointments in total to obtain a 3D volume.

### Semi-automatic segmentation results

At visual inspection, we estimated that 57% would require no or minimal manual refinements, 35% required significant corrections of part of the segmentation, and 8% would need major changes or were judged difficult due to poor visibility. This assessment translated into the time spent on the manual edits that we estimate to be between 0 and 10 min for the first group, less than 45 min for the second and up to 2 hours for the more challenging cases. A median time of around 9 min was measured; the mean time spent by segmentation is significantly higher due to the most difficult cases and was around 20 to 30 min. In addition to this time, 5 to 10 min were spent per case placing the landmarks and setting up the automatic segmentation. The degree of overlap between the automatic and edited segmentations was associated with an average Dice score of 97%. The average symmetric surface distance (ASSD) between pre- and post-manual refinement was 0.14mm (for a 0.6mm image resolution) with 44% of the cohort having an ASSD inferior to one tenth of the voxel size (<0.06mm).

The need for manual refinement was directly related to the quality of the 3D US volume, when the image captured an unencumbered view of all borders of the fetal face, no manual refinement was required. Most of the required manual refinements were associated with missing regions of the face. The regions with low contrast in the images are poorly segmented and the peripheral areas of the face more often required manual editing.

### Growth curve model

**Figure 1:**
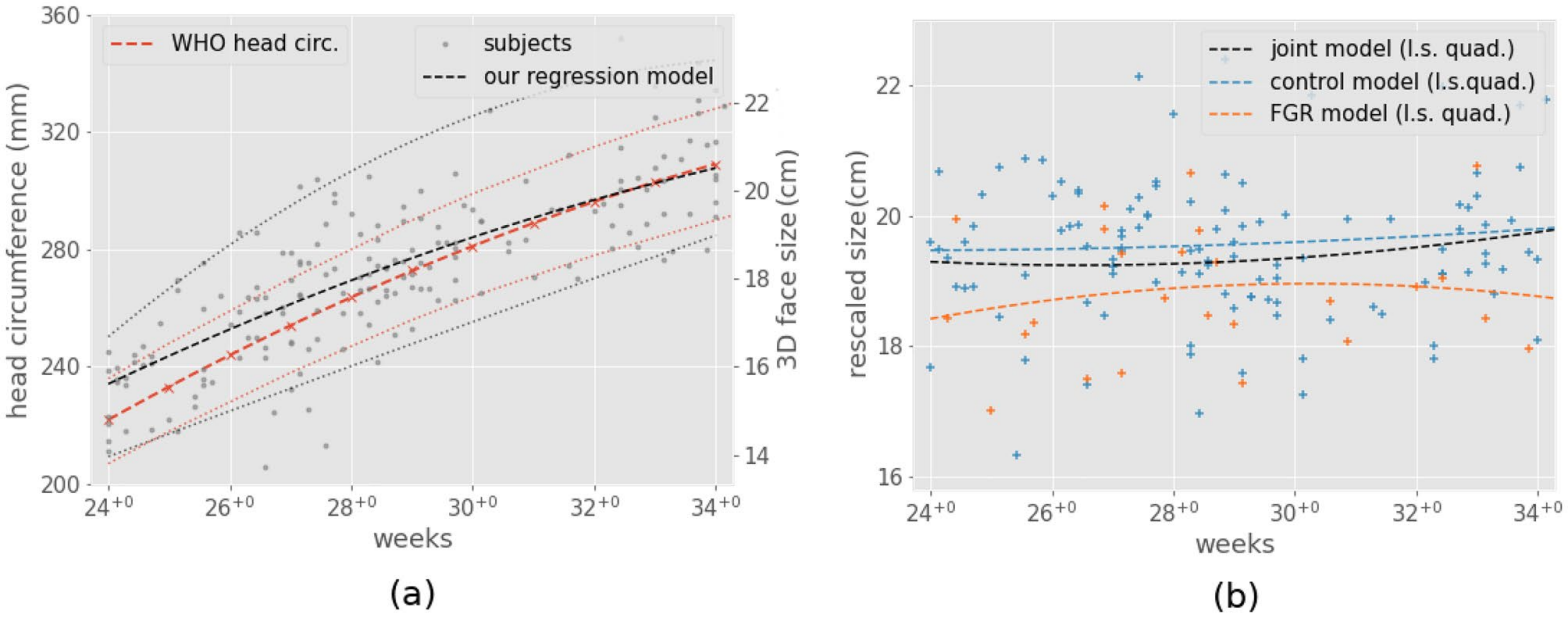
Size measurements of the fetal face. (**a**) face and head sizes with respect to age before rescaling: our regression model (black), reference model for head circumference from Kiserud et al.^29^ (red). The quadratic growth curves are estimated using quantile regression (medians are represented by dashed lines, 5th and 95th percentiles by dotted lines). (**b**) face size measurements after rescaling. Regression models are shown for AGA cases (blue), FGR cases (orange) and both combined (black).

The estimated growth model of the fetal face allows correction for age-related differences prior to the statistical shape analysis. The scale of the segmentation meshes was measured using an approximation of the face area based on the first two modes of the principal component analysis (PCA) on the points of the reconstructed facial mesh. We compared the resulting growth curve estimation from our dataset to published growth curve for head circumference^29^(Fig. 1a).

The two curves are almost proportional, describing a similar growth rate, and would lead to a similar rescaling. The difference between both scale factor would be on average less than 1% and always inferior to 3% (maximum at 34 weeks gestation). The variability is higher for our measurement, probably due to the noise associated to the segmentation and automatic metric. We chose to rescale the meshes using the growth curve estimated from our dataset because it describes the face size directly rather than head size.

After size correction, using the growth curve, the average face size did not show any visible correlation with age (Fig. 1b). When comparing the FGR cases (orange line) to the AGA cases (blue line) a consistent difference is visible between the two groups.

### Statistical shape model of the fetal face

**Figure 2:**
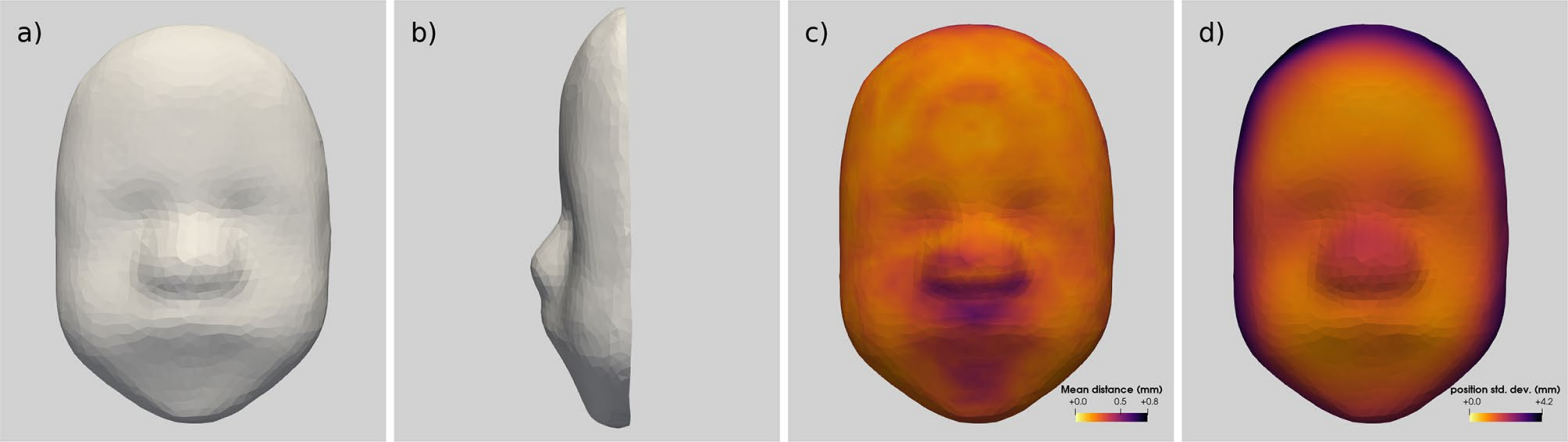
(**a**, **b**) Mean template of the fetal face, the template is warped by subject-specific deformations to match each individual morphology. (**c**) Average reconstruction error at each point, measured as the distance between the reconstructions and the original models. Small errors are yellow/orange and larger errors purple. d) Shape variability measured using the standard deviation of the mesh node positions over all of the reconstruction. Purple denotes the areas with the highest variability and yellow/orange the most stable areas.

The estimated morphological reference and shape variability in the population are both shown in Fig. 2. The reference shape, also called template, represents the anatomical mean shape of the population (Fig. 2a,b). As a results of averaging, the template morphology is symmetrical in appearance with smooth facial features and a loss of definition of fine detail.

The reconstruction error is relatively small with an average distance between the model mesh nodes and the original segmented surface of 0.29mm (highest = 0.79mm at a single point). Only 5 cases have an average distance superior to 0.4mm, these cases are associated with extreme phenotypes with overly large or small faces. The reconstruction error is partially associated with the smoothing of small noisy defects from the segmentations but is more pronounced in the areas with more fine details such as the lips and the nose (Fig. 2c).

The shape variability captured by the model (Fig. 2d) is one order of magnitude higher (2.10mm in average) than the reconstruction error, showing evidence that the shape model is able to capture most of the variation between subjects. The highest variability of the model is at the edge of the mesh while the forehead is the most stable part .

Some of the variations could be related to the pre-processing steps: the segmentation and mesh cutting may increase the variance near the edges while the iterative closest point (ICP) rigid registration may artificially reduce the variability in the central areas. In addition, any size differences would be more apparent near the edges than closer to the centre. To a lesser extent, some variability is noted around the nose, the nasal arch and the eyes. This effect could be associated with small differences in eyes and nose position, size or with more local differences in their shape. Less variability is measured on the forehead, other than the upper-edge, and around the mouth despite the mouth being a potentially well-defined anatomical features.

**Figure 3:**
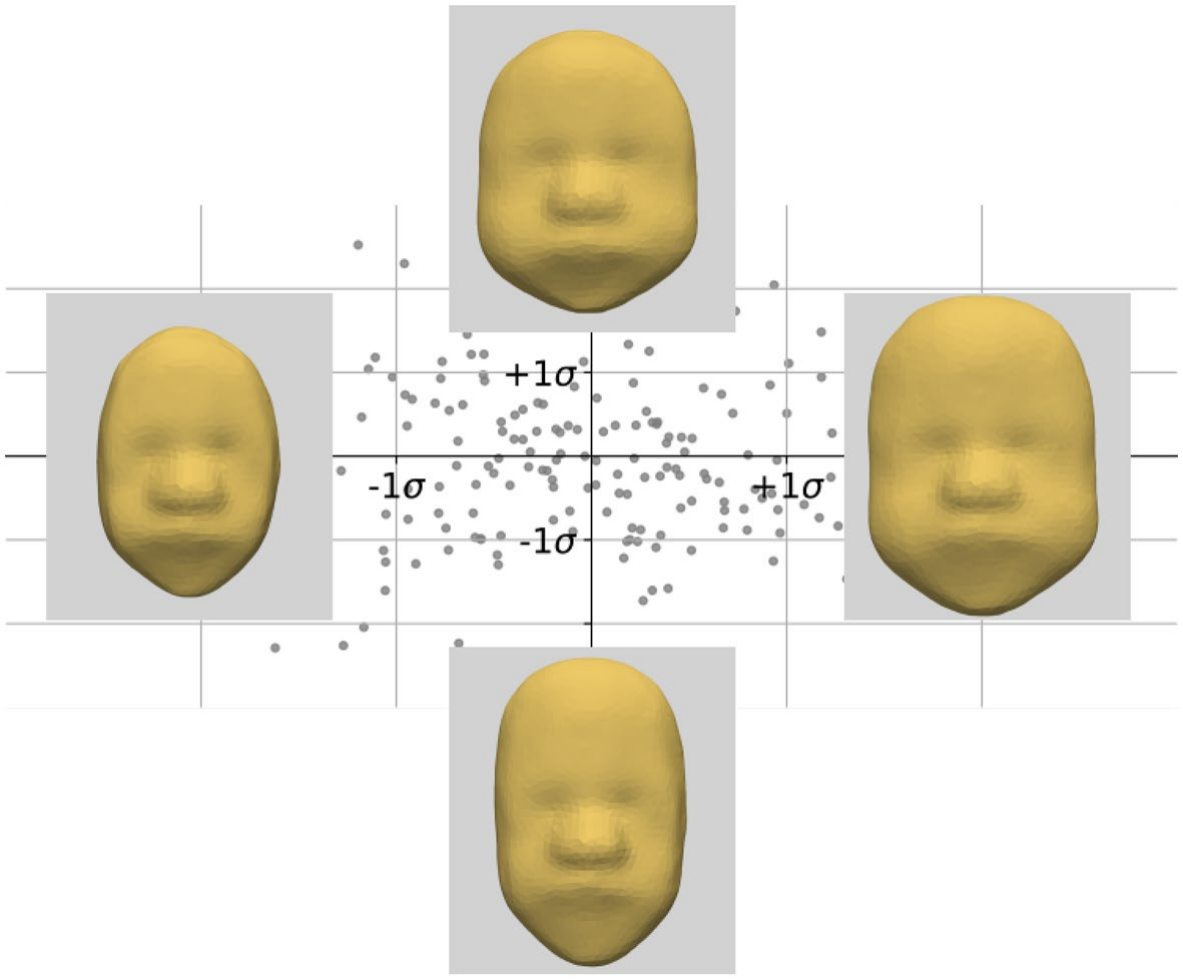
PCA projection of the first two modes of the SSM model. The first mode is represented horizontally, the second vertically. Each grey dot represents one subject. The faces illustrate the model at −/+ 2 standard deviations.

The main axes of variations computed using a PCA decomposition of the momenta are shown in Fig. 3. The first two modes capture 41.5% and 7.6% of the total variance respectively. 26 modes are needed to describe 90% of the total variance. The first mode (represented horizontally) shows the size of the face, specifically facial width to be the most important contributor to shape variability. The overall shape of the face varies between an oval to a more ‘squared’ facial shape. In this mode, while the whole face gets larger the centre of the face gets relatively smaller; with eyes getting closer and the nose getting a bit flatter.

The second mode (represented vertically) illustrates a strong emphasis on the width of the cheeks and forehead length with negative values showing a trend towards a longer thinner face. The second mode is positively correlated with age (Pearson’s correlation coefficient test: $r = 0.28$, $p < 0.001$). A similar correlation with age is also noted with the 3^rd^, 5^th^ and 6^th^ modes. We can note that the first mode is not correlated with age ($r = 0.009$), reflecting the correction for age-related size changes.

### Gestational age-related changes in the control population and growth restriction

**Figure 4:**
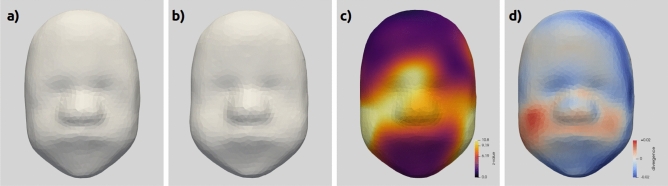
The effect of age on fetal facial morphology. (**a**, **b**) Model of the average morphology at (**a**) 24 weeks and (**b**) 34 weeks of gestational age. (**c**) Illustration of the local z-values assessing the effect of gestational age on morphology. The colour bar is normalised to show levels corresponding to *p*=0.5 (purple), *p*=0.1 (orange) and *p*=0.05 (yellow). (**d**) divergence of the deformation vector field (momenta).

Changes associated with GA were estimated using linear regression on the momenta. These changes complement the size change that was modelled in the previous section using the scalar growth curve. Figure 4a and b shows the model of the average face at 24 and 34 weeks. Advanced GA is associated with wider fuller cheeks, flatter foreheads, deeper eyes and slightly bigger noses compared to earlier GA.

Figure 4c is the projection on the face of the test statistic (z-value) used in the permutation-based likelihood test to assess where the age-related changes can be considered significant. The z-values are computed at each of the deformation control points and then represented on the template surface. Here the z-values is higher on the cheeks and in the centre of the face, especially around the nasal arch. This age effect was associated with a *p*-value p<0.001 for 10000 random permutations.

The divergence of the deformation (momenta) vector fields on the surface (Fig. 4d) can help visualize the changes by focusing on the local volume changes. We see here an expansion of the cheekbones, the posterior aspect of the jaw, and around the nose, and a contraction of the chin, the left side of the face and near the eyes.

### Describing fetal growth restriction with a shape model

**Figure 5:**
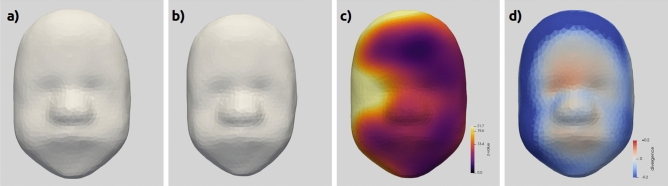
Differences associated with FGR: (**a**) average control face, (**b**) average FGR face (normal diagnosis only), (**c**) group comparison z-value assessing for the effect of FGR on the morphology, the colour-bar ticks are normalized to show values corresponding to *p*=0.5 (purple), *p*=0.1 (orange), and *p*=0.05 (light yellow) for the t^2^ statistic. d) divergence of the deformation vector field (momenta).

Cases of fetal growth restriction (FGR) without any underlying genetic condition (n=18) were compared to the normal controls (n=117). The main difference is a difference of size with smaller average for the FGR cases (Fig. 5b) than for the control cases (Fig. 5a). The facial features do not show any other clinically meaningful difference between the two groups.

The statistical significance of these differences is assessed using two-sample Hotelling’s t^2^-test on the deformation parameters at each control point (Fig. 5c), highlighting differences over the mid-face with larger differences asymmetrical differences seen on the right side at the level of the eye. The *p*-value estimated using 1000 random permutations was equal to p=0.008.

The visualization of the divergence field (Fig. 5d) confirms these observations. A strong contraction in a ring all around the face, the effect is stronger on the right side. The centre of the face is on the contrary quite preserved, with an hint of a small expansion near the top of the nasal arch.

### Variability within a normal face

Descriptive statistics, such as mean, variance or PCA modes, are useful to describe the population variability and the differences associated with clinical variables such as gestational age or diagnosis. In this section, we propose to use the shape model to inform us about a specific subject, and its deviation from the control cohort. Compared to the statistical tests of the previous sections, we do not focus here on the evaluation of a specific hypothesis but on a qualitative description of the differences. Our approach is a generalization, to morphological differences, of the reporting of z-scores (also called standard scores). The standard shape scores are computed at each point on the face based on the momenta values. The shape distribution parameters (mean and covariance) are estimated using the control AGA samples only. We propose two scores.

The first one, the *deformation score*, is based on the Hotelling distribution previously used, and takes into account the full 3D deformation vectors. The second one, the *orthogonal score,* only considers the projection of the deformation orthogonally to the surface. The second score is more directly interpretable as it describes changes in and out of the surface, the first one however can capture tangential morphological differences.

**Figure 6:**
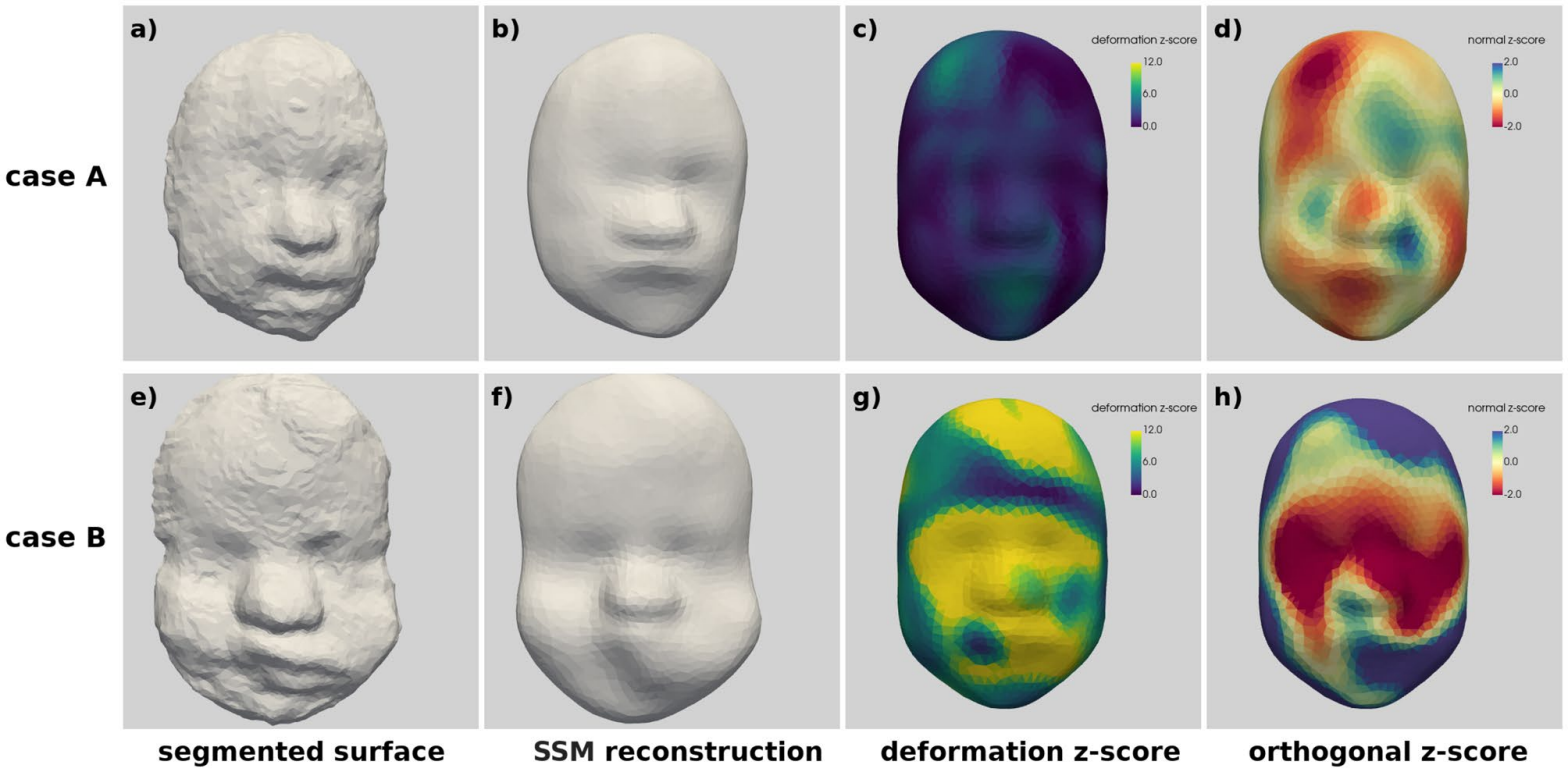
Two cases with normal diagnosis: case A top row and B (the most “abnormal normal”) bottom row. From left to right: original mesh, model reconstruction, deformation z-score, orthogonal z-score.

We show in Fig. 6 the results for two normal fetal faces. Case A, selected randomly, is a 24+3 gestational week fetus. Case B is the normal case with the highest average deformation score (32+3 weeks of gestation). In case A, the deformation score (Fig. 6c) shows small differences on the right upper lateral aspect of the forehead but overall no meaningful difference throughout the face. The orthogonal score is more variable over the face with high and low areas, the negative values on the upper-left demonstrate that the this fetal face is characterized by receding forehead in this area (Fig. 6d). The deformation score for case B (Fig. 6g) demonstrates large differences at upper middle of the forehead, across the eyes and on the left aspect of the mouth and lateral border of the chin. The orthogonal score (Fig. 6h) shows that these differences in the forehead and mouth/lateral aspect of the chin represent values above the mean and the differences across the eyes and mid-face represent values below the mean. When inspecting this face visually, the lower half of the face appears to be slightly distorted as though the lower right aspect of the chin is pressed against something, distorting both that side of the mouth as well as the contralateral side which may have caused the differences identified and further highlights the importance of high-quality US volumes with clear borders.

## Discussion

The aims of this study are to propose a shape model of the normal population and its variability, and to showcase a morphometric approach that objectively describe fetal face features and could assist the diagnosis of complex fetal phenotypic variants.

### Segmentation algorithm

 The analysis of a fetal face from a 3D volume generally require several manual processing steps, from the image acquisition, to the landmark annotation, or the image segmentation and creation of the 3D model. These steps are time consuming and challenging to automate due to imaging noise, fetal movements, low soft-tissue contrast, and occlusions. Fast and reproducible processing methods are required to conduct large studies and to develop clinical tools.

The introduction of two additional steps to our segmentation algorithm (4 manual landmarks and monogenic signal map) improved the performance compared to our previous work^26^ reducing the need for manual refinement. This improvement enabled the processing of a large number of 3D US and the reconstruction of a population of 3D surface models of the fetal face. In order to allow the inclusion of the maximum number of subjects within this work we included cases requiring some manual refinement. It is however unrealistic in clinical practice to expect clinicians to undertake manual refinement of the segmentations prior to analysis.

Recent algorithmic contributions in fetal ultrasound image processing especially using deep learning^16,30,31^ are promising, but the development of a reliable segmentation of the 3D fetal face remains a challenge and publicly available data-sets very limited^32,33^. The validation of our algorithm and the comparison to other existing approaches^14,16^ would benefit from experiments on an independent set of images, in particular if we want to extend the study to more diverse settings. For example, at earlier gestational ages,the signal to noise ratio would be weaker and smaller fetuses would mean lower resolution. The effect of segmentation variability induced by the use of different segmentation algorithms, imaging methods, and manual edits would also need to be assessed.

### Growth model

The analysis of the morphological variability of the fetal face cannot ignore the growth associated with gestational age. The use of growth-curves and age-specific morphological references is an established standard in many clinical applications but shape modelling approaches generally consider these changes in a latter stage of the statistical analysis^20^.

We proposed in the work to include an initial model to approximate these changes using an isotropic size increase. This original approach allows the 3D shape model to describe the inter-subject size variability and to fully characterize subtle age-related changes without being dominated by the large differences between less and more advanced gestational age cases that could hinder the estimation of the statistical model and the analysis of the results. This approach is directly generalizable to other settings where size is an important factor and inter-subject size differences should be accounted for.

In our case, we showed that the reference growth rate could be directly estimated on the included sample or based on established measurements. In particular, we found similar results with a model based on facial surface area or on tabulated fetal head circumference. The highlighted size differences between AGA and FGR cases were an additional motivation to look, using computerized morphometry, for more extensive information about the associated morphological features or development delay that could be associated with this diagnosis.

### Shape reference

In the last decades, statistical shape modelling has seen many potent development to describe population of 3D objects. In particular, morphable face models have been used to describe facial dysmorphisms^19,20^ with potential applications such as the early detection of genetic syndromes^21^. The quantitative analysis of the fetal face has however been limited by the difficult reconstruction of the 3D surface^17^.

We previously demonstrated the feasibility of using landmark-free diffeomorphic morphometry to model fetal faces^24^. We show here that this approach is scalable to a large number of cases, and that the model is able to reconstruct every face with high accuracy at a relatively fine scale. In particular, the reconstruction error is small in comparison to the shape variability captured.

The model is also generative and the shape space can be used to sample synthetic cases that are realistic, as shown by the visualization of the 2 first PCA modes. The unsupervised statistical model is able to describe some localized shape features (shape and size of the nose, position of the eyes, etc.) and more global change of the overall shape of the face (oval, square, etc.) that are difficult to quantify otherwise. The correlation of PCA mode 2 with age illustrates some potential application of the these descriptions to analyse clinical correlates. As such, the model, using the mean template and the distribution of shape vectors, appears to be a valid reference point for comparison.

### Age-related changes and fetal growth restriction

Using multivariate statistical analysis of the shape model parameters, we highlighted morphological changes associated with age that were complementary to the uniform size increase. We described wider cheeks and more delineated face features of the fetus, not to our knowledge previously described in detail. These changes were assessed to be significant in our statistical model. To the best of our knowledge, these gestational age-related changes have not been previously described in details in the literature, however analysis of fetal facial bone growth using post-mortem CT scans^34^ demonstrated that an increase in the anteroposterior mandibular diameter, maxillary width and bimandibular distance is strongly correlated with an increase in gestational age. A weaker correlation to zygomatic bone growth was also identified. These findings are consistent with the age-related differences found in our analysis.

Similarly, we also showed significant differences associated between AGA and FGR cases. FGR diagnosis was associated with a smaller face but with no other facial feature was apparent in our analysis, aside from an slight asymmetrical difference at the level of the right eye.

It is difficult to compare the effect of gestational age and the differences AGA/FGR. First, age-related size differences have been partially accounted for by the growth curve model while no uniform scaling have been applied depending on the FGR diagnosis. And, overall, even if some similarities with the age-related changes could be suggested (on the cheek width or jaw line for example), no clear face features significantly differ between AGA and FGR cases and the global differences do not match changes that would be associated with any gestational age gap. We should however keep in mind that it is difficult to guarantee that the model is able to represent as accurately FGR cases than AGA cases due to the size difference.

Another hypothesis is that the suggested differences in the fetal cheeks may represent a degree of fetal malnutrition with the loss of buccal fat and sharply defined jaw and chin that can be seen in FGR and is a key part of the clinical assessment of nutritional status (CAN) score to assess newborns^35^.

Our sample size is also relatively small and the localization of the difference is not the main outcome of the statistical analysis. For example, the asymmetrical difference may be explained by the capturing of the original 3D ultrasound volume; 8 of the 18 cases were captured with the fetal face deviated to the right resulting in slightly less clarity in the right side of the fetal face, 2 cases were slightly deviated to the left and the remaining cases were positioned centrally. This may have contributed to the asymmetrical difference noted in the analysis. Validation of these explanatory results on an independent dataset would be valuable.

This analysis also higlights the difficulty in visualising, identifying and characterising the morphological differences at the very edge of the facial mesh.

### Shape z-scores

The analysis of individual cases provide one illustration of potential use of the models to extract, from the three-dimensional ultrasound, more detailed information about the facial morphology than the conventional 2D US analysis. Were this technique to be used in clinical practice in the future, we envisage that it would be used by clinicians to describe analyse and describe cases in which an underlying syndrome is suspected but in which the diagnosis remains elusive despite invasive tests, rather than being used as an unrestricted diagnostic tool.

The high scores of case B (above) demonstrated the limits of this approach. From its high scores, this subject seems to be abnormal or even an outlier. It is normal for some subjects to be on the extreme of the distribution. The surface mesh do not reflect the uncertainty of the segmentation in regions were the contrast is low and the face border poorly visible. In this case, it is possible to precisely locate the face limits in an area near the mouth. Moreover, the bump on the forehead is arguably a segmentation error. The head is very near the edge of the US field of view, and this sharp edge tends to deform the image and pull the segmentation at the expense of the correct head border.

### General limitations

The methodology that is described is not yet applicable to clinical practice, chiefly because of the amount of time required for segmentation and analysis (1 to 1.5 hour for one case with, in average, around 30 min of manual work). Also the study only includes two imaging centres and the segmentation process has been centrally and uniformly conducted. A more scattered clinical deployment would lead to more heterogeneous results.

A single surface mesh is a very simple representation of the morphological information available in a 3D ultrasound. It is only a summary of the interactive and dynamic signal visible during the examination. The selection of one volume to describe the entire face is already a limitation. In addition, meshing and smoothing loose fine, and less visible features.

A related limitation comes from the fact that we are trying to model the shape of soft tissues in a complex environment. In particular, the face is deformed by contact with fetal limbs, or maternal tissues, and these contacts are not only not modelled but also hidden to the final analysis based on the surface reconstruction only.

### Perspectives

In this work, we describe a semi-automatic algorithm to build a shape model of the third trimester fetal face from 3D ultrasound and demonstrate how it can objectively describe morphological features and gestational-age related changes of normal fetal faces. This study represents an important step for objective assessment and characterisation of normal fetal face development and variability in the third trimester. Using these templates, future work will allow the model to be assessed for its discriminatory ability in the diagnosis of congenital anomalies and dysmorphic or syndromic facial features.

## Methods

### Patient population and image acquisition

A prospective cohort study of women attending for a fetal medicine scan between 24-34 weeks gestation was performed. Recruitment took place at 2 tertiary fetal medicine centres between January 2019 and May 2021. The study was approved at both units (REC approval 18/WM/0370 by the National Research Ethics Service Committee West Midlands – Edgbaston Research Ethics Committee and 579/2019/OSS/AOUPR by Comitato Etico dell’Area Vasta Emilia Nord and written informed consent from study participants was obtained.

Diagnosis information was extracted from clinical reports, and confirmed after 12 months follow-up. FGR diagnosis is based on the Delphi criteria^36^.

**Figure 7:**
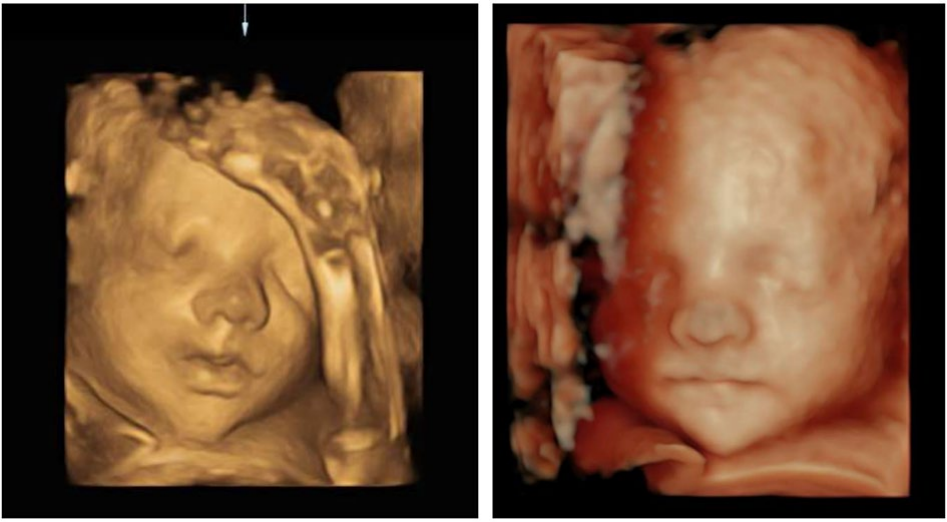
Two 3D US volumes collected from the same participant during the same appointment. The volume on the right was selected as the facial border were clearer and there was no fetal limb in contact with the fetal face as seen in the left image.

Participants for whom it was not possible to collect a facial volume between the 24 and 34 week of gestation were excluded. Volumes that did not show the complete face or in which the fetal position did not allow for good-quality segmentation were also excluded from analysis. When several volumes were available for the same patient, the best one was subjectively selected based on image contrast, clarity and clearness of the facial borders and facial field (see Fig. 7).

**Figure 8:**
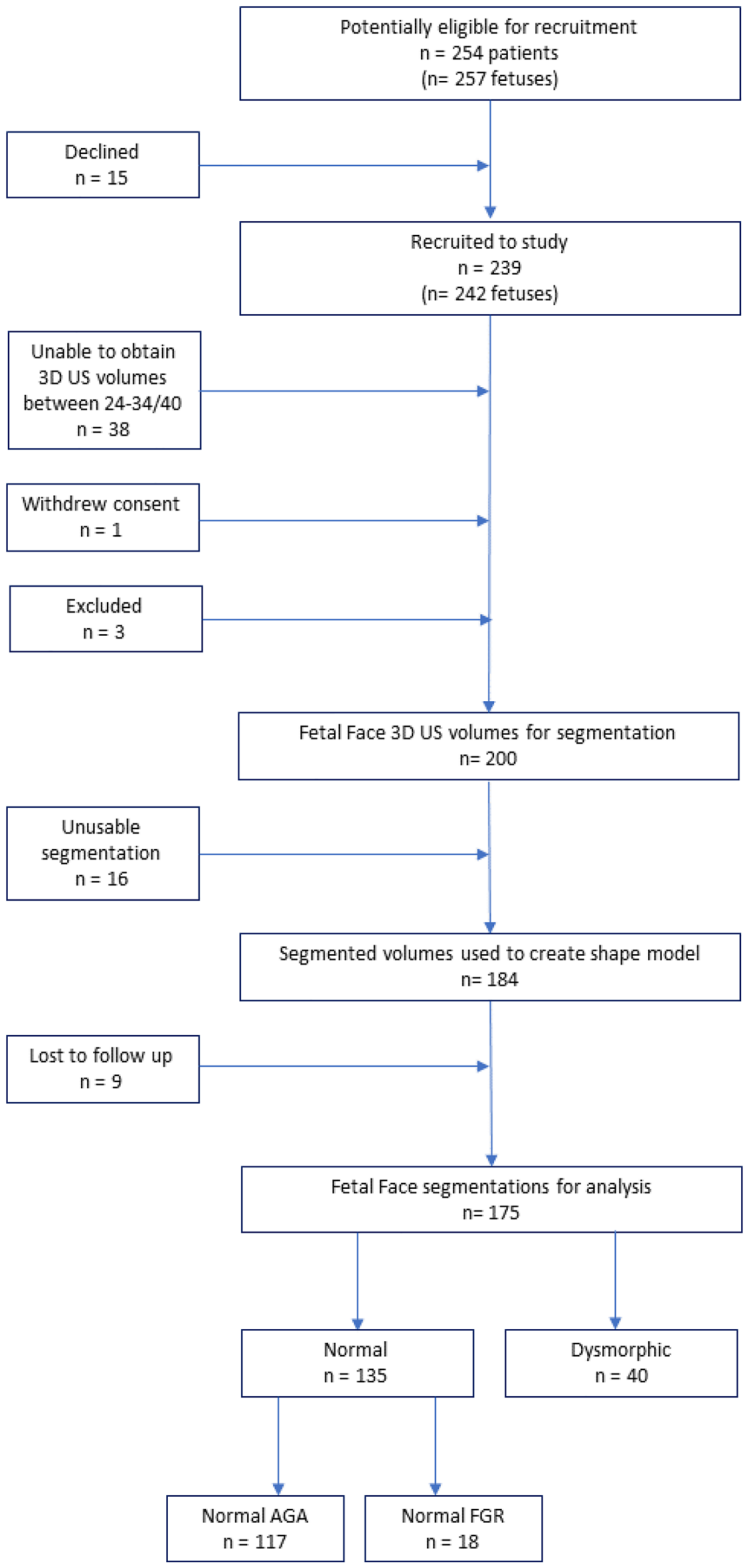
Flowchart of recruitment, 3D US volume acquisition, segmentation quality and final numbers for analysis.

Participants with incomplete follow up data or those with an uncertain diagnosis were also excluded from analysis. Finally in this study, we focus on patients without any diagnosed facial dysmorphism. The inclusion process is described in Fig. 8.

The ultrasound images included in this study are 3D static acquisitions obtained in the midsagittal plane of the fetal face, using the highest resolution acquisition mode with a low frequency probe (4-8 MHz) using a Voluson E8 or E10 (GE Healthcare) or a Hera W10 (Samsung) ultrasound machine. The volumes were aligned in the multiplanar view along the x, y and z axes and subsequently exported as 3D images.

Acquisition and processing of the patient data were carried out in accordance with clinical guidelines and data protection regulations.

### Semi-automatic segmentation workflow

**Figure 9:**
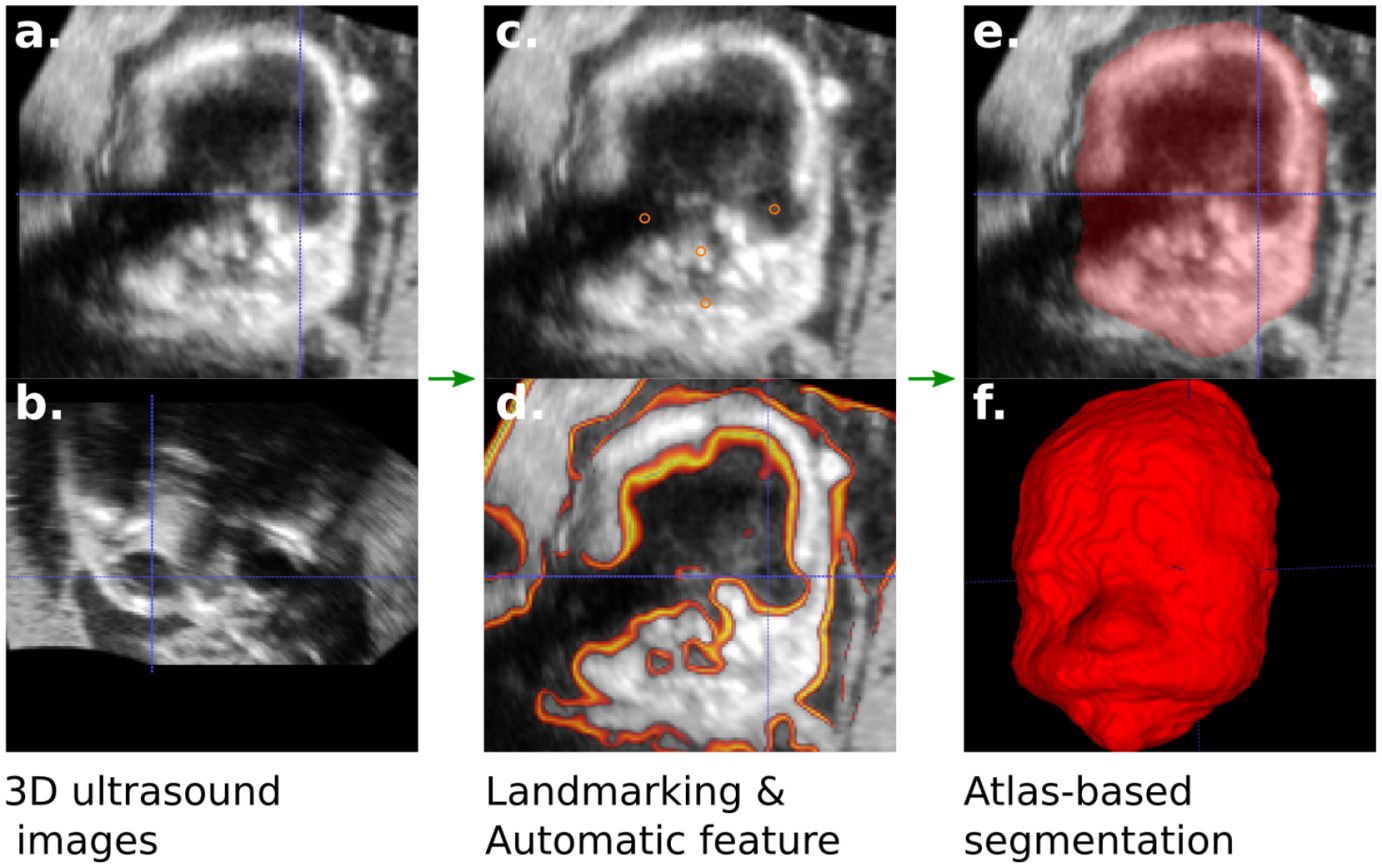
Overview of the atlas-based segmentation algorithm steps: (**a**, **b**) 3D US volume (**c**) 4 manual landmarks (eyes, nose, midpoint of the lips) (**e**) monogenic signal map (**e**) segmentation mask f) 3D surface mesh.

Several steps are required to create a 3D reconstruction mesh representing the face shape from the ultrasound volume (Fig. 9a,b). We previously adapted the atlas-based segmentation algorithm developed by Zuluaga et al. (2013)^27^ to the fetal faces with good result^26^ however it still required significant manual refinement.

We introduced two further improvements to the algorithm in this work. Firstly, the images are initially rigidly aligned using four manual landmarks (eyes, noise, midpoint of the lips, see Fig. 9c) to correct for head position. Secondly, the image registration similarity, key element of the segmentation algorithm, includes an additional term designed to guide the segmentation with US-specific signal properties. This term is defined by the normalized cross-correlation between monogenic phase asymmetry map computed for each image (Fig. 9d). The asymmetry map is defined by:

$$R(x) = \frac{\max (0, |f_o(x)| - |f_e(x)| - T)}{A(x) + \varepsilon }$$

where $f_e$ is the radial part of the image signal, $f_o$ is the odd part, *A* is the local amplitude, and *T* a user-defined threshold between 0 and 1 (that we set to 0.5). We refer to Bridge’s introduction to monogenic signals^37^ for more details.

Monogenic signals have proven to be useful to capture edge features in clinical ultrasound images^38^. A similar idea has been used by Qiu et al. (2017)^39^ who computes the phase congruency maps of ultrasound volumes to drive the registration of an atlas-based segmentation of neonatal cerebral ventricles.

Every resulting segmentation was visually checked and manually edited in ITK-snap (itksnap.org) to correct for mis-segmentation and small missing parts (Fig. 9e). If the boundary could not be reasonably estimated, the volume was excluded. This step is crucial to correct for errors in the automatic segmentation caused by image artefacts or obstruction of the face and to select the best image for each subject. A closed surface mesh was then exported (Fig. 9f).

### Mesh processing and size normalisation

To assess meaningful features in the shape analysis, the 3D meshes have to be relatively regular and all orientated in the same position. This is done in two steps. Firstly, remeshing is performed using pygalmesh software^40^ to optimise the cell shapes and provide a more uniform cell density. This step can also be used to control for the average number of mesh elements, an important factor in the trade-off between computational cost and resolution. We set the target triangle radius to 2mm and the facet distance to 0.1mm, leading to approximately 6700 cells per mesh. Secondly, meshes are rigidly aligned using the 4 previously defined manual landmarks and automatic iterative closest point registration (ICP). Meshes are then automatically cut based upon a predefined plane and the orientation of cell normals.

An important question to address is the size normalization. The dominant change between 24 and 34 weeks is associated with the fetal growth and, if unaccounted for, this size variability can hide more subtle, interesting, and potentially clinically significant differences, in the estimation of the statistical shape model. The growth can be, in a first step, approximated by an isotropic size increase. Morphometric measurements are often normalized for organ or body size (see^41,42^ for example), however we are here interested in modelling the change with age and size differences are not only caused by the fetal growth but also by inter-subject variability. Rescaling the geometries through similarity registration or using measurements of the fetal face (such as head circumference) would erase this information despite it being clinically relevant (for the study of fetal growth restriction (FGR) for example). We chose an intermediate approach where the faces are rescaled according to a growth model giving the average size at every gestational age. Younger cases are enlarged, and older cases are shrunk towards an average size corresponding to the median age in our population (29 weeks).

In absence of ground-truth size measurements (such as a manual head circumference measurement), we chose to use the square root of the area of the face estimated using the variance along the two principal directions of the point cloud. This point cloud-variance metric has been chosen rather than a more direct measurement of the mesh surface area because of the strong, albeit consistent, bias introduced by the mesh processing steps on the direct measurement (the mesh exported from the segmentation is a close surface with a noisy back side while the pre-process mesh only preserves the open surface corresponding to the face, this process reduces the total mesh surface by around 30 percent). The selected measure is also fully automatic, fast and easily adaptable to various settings.

The growth model is defined by a quadratic curve fitted by quantile regression to this surrogate size in our complete population (cases with and without pathologies). This model will be compared to tabulated growth curves for head circumference^29^.

### Statistical shape modelling

The shape analysis approach used in this work relies on the Deformetrica framework^25^. In this framework, geometrical objects are compared though the dense deformations of space that can transform one shape into another. The aim is to enable the comparison of morphologically distinct objects without requiring any point correspondence. The space of valid deformations is parametrized by infinitesimal displacements, called momenta. These momenta are defined in the whole 3D space and belong to a reproducing kernel Hilbert space (RKHS) built using a Gaussian kernel with fixed width and a set of control points. The advantage is that, by construction, the deformations are diffeomorphic, fully parametrized by 3D vectors defined on the set of spatial control points, and that linear operations, and usual Euclidean statistics, can be used in this space. The position of the control points is optimized to efficiently sample the ambient space occupied by the 3D surfaces.

**Figure 10:**
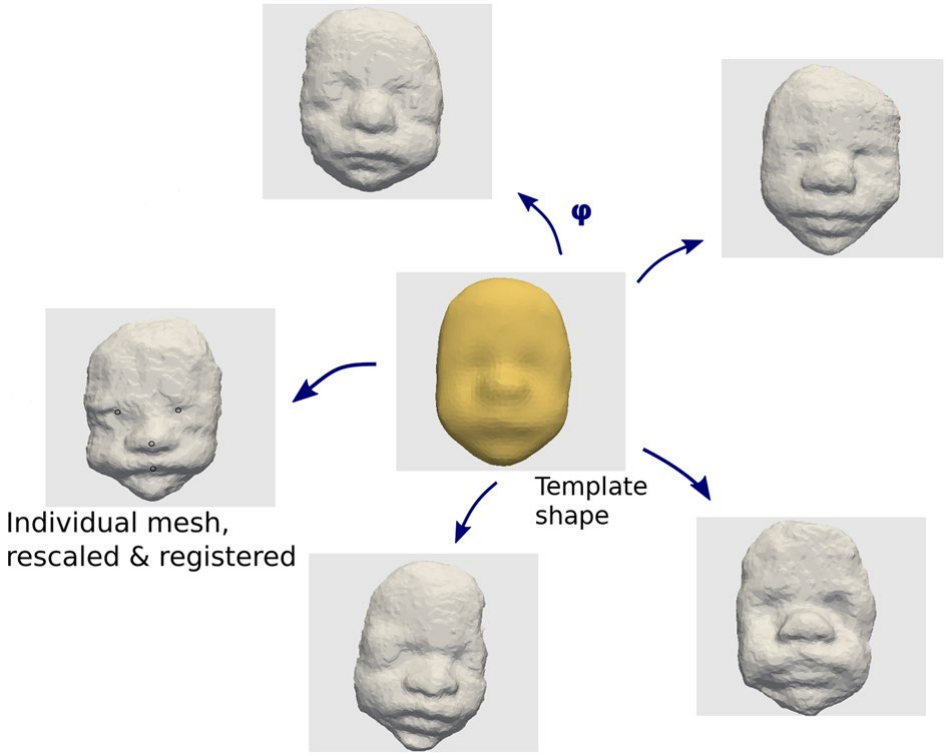
Schematic representation of the statistical shape model: template shape to individual cases/deformations.

This framework is then used to build a statistical model of the shape population. The most common approach is called atlas and is constituted of two main components: a reference shape called template that is representative of the anatomical mean shape in the population, and individual deformations that map this template to each individual shape and capture the morphological variability in the population (Fig. 10). Once the atlas (the template and the individual deformations) has been estimated, the statistical analysis is conducted directly on the deformation parameters (the momenta vectors defined on every control points).

### Shape atlas estimation

The shape model is controlled by two main parameters: the resolution $\lambda _W$ that controls the scale at which the objects are described, and the stiffness of the deformations $\lambda _V$ that controls how smooth the deformations between subjects will be^43^. It is possible to intuit reasonable values for these parameters based on the regularity of the objects under study. To inform our choice, we conducted preliminary experiments to test for a range of values between 3mm and 20mm (using only the subset of normal cases to speed up the process). The best $\lambda _W$ value was selected using the Akaike information criteria (AIC) to balance for the increase of number of deformation control points when the resolution decrease. The average reconstruction error was used to select $\lambda _V$.

No major difference was observed for $\lambda _W \in {5: 10}$ with a minimum at 7mm. Smaller values were unable to be evaluated due to RAM limitations. We then set $\lambda _W = 7$. The reconstruction error was monotonically increasing for $\lambda _V \ge 5$, but slowly for $\lambda _V \in {5: 9}$ and with numerical instabilities for smaller values (not converging for $\lambda _V= 4$ and poor result for $\lambda _V= 3$). As a compromise, we chose to set $\lambda _V=$ 7mm.

The initialisation of the optimization could also play a role; mainly, the initial template shape, and the position of the deformation control points. To address these two questions, we iterate over several model estimations focusing on these aspects first. Designing an approximate average face is not too difficult as there are no major topological differences between subjects; from a random initialisation (one average-sized subject), a first template is estimated and then manually smoothed and remeshed. The second optimization run focuses on control point positions, with a fixed template. Points associated with almost no momenta variance (less than 0.01% the maximum) were then filtered out in order to remove the ones that were outside the space covered by the faces and speed up the model estimation.

### Statistical analysis of the momenta

The atlas model and the compact parametrization of deformations by momenta facilitate the study of morphological features and of their relations to external independent variables. However, the statistical analysis is still challenging. One of the main challenge lies in the dimensionality of the data, even if it has been strongly reduced compared to the original surface meshes, it is still too large for naive statistical approaches and visualization. For reference, our final model involved 412 control points, by consequence the shapes are characterized by *D *= 412$\times$3 = 1236 parameters. We summarize here the key elements of the main statistical methods that will be used to analyse the momenta distribution and their relation to clinical variables.

Principal component analysis (PCA) is used to describe the shape distribution using a reduced number of modes. Singular value decomposition is performed on the momenta matrix and the first modes are extracted. It is a useful tool for visualization and interpretation purposes as it gives a low-dimensional representation of the shape population. It can also hint towards important variability factors that could be explicitly considered in the model. For instance, size is, in many applications, the first or one of the first modes^44^.

Multivariate discriminant statistical testing is used to assess the existence and localization of a correlation between clinical variables and morphological features. A statistic is computed at each deformation control point and a permutation scheme is used to estimate an empirical distribution of the maximum of this statistic under $H_0$. This approach gives a non-parametric *p*-value that is both a global assessment and an indicator of the localization of the effect. This approach avoids the problem of multiple testing^45^.

A specificity of our work is that the statistics, computed at each control points, are also multivariate. Indeed, we need to account for the 3 directions of the momenta vectors. In this context, we use the Hotelling t^2^-statistic to assess the difference of mean value between two groups. If the independent variable is continuous (such as age for example) or if multiple variables are jointly considered (multiple diagnoses and age correction for example), we used log-likelihood testing for multivariate linear regression^46^. We can note that this approach is equivalent to the Hotelling test when there is only one binary variable . Unless specified, *p*-values are calculated using N=1000 random permutations and significance level is chosen to p=0.05.

### Shape-related z-scores and assessment of the deviation from the control population of individual subjects

We propose to use *standardized shape scores* to describe and visualize the deviation of individual morphologies from the population distribution characterized by the statistical shape model. This approach is a generalization of the reporting of a z-score (also called standard score) in the case of a scalar variable. Standard scores could be computed directly on the momenta parameters at every control points; to get a more visual and detailed description we propose to compute them at each cell center of the template mesh instead. The momentum vector *m* for a subject *k* at any point *x* in space is directly computed from these momenta parameters $(a_i)^k$ defined at each control point $(c_i)_i$ (see Durrleman et al. (2014)^25^ for more details):

$$m^k(x) = \sum _i{K(x, c_i)a^k_i}$$

Parameters $a^k$ for a new subjects are estimated through registration to the template face.

We propose two scores: the *deformation score* based on the Hotelling statistic for 3D vectors, and the *orthogonal score* for changes locally normal to the surface. We first compute the average $\bar{m}(x)$ and the empirical covariance $\bar{\Sigma }(x)$ of the momenta vectors on the set of control subjects used to build the model. Then: we define at each point the *deformation score* of subject *k* by

$$z^k_d = (m^k - \bar{m})^T \bar{\Sigma }^{-1} (m^k - \bar{m})$$

The *orthogonal score* uses the local surface normals *n*(*x*) and can then only be computed on the template surface mesh. We define $\bar{\sigma _n}^2 = \frac{1}{N-1} \sum { ((m-\bar{m}) \cdot n)^2 }$ the empirical standard deviation, on the control samples, of the projection of the momenta along these normals. The orthogonal score writes

$$z^k_o = \frac{(m^k-\bar{m}) \cdot n}{\bar{\sigma _n}}$$

A signature score, similar to the *orthogonal score* has been used for paediatric patient by Matthews et al. (2021)^20^. The changes associated with this second score are more directly interpretable but do not take into account the complete deformation vectors and can overlook more complex morphological differences.

If we assume the momenta vector *m*(*x*) follows a multivariate Gaussian distribution $\mathcal {N}(\bar{m},\bar{\Sigma })$ (only true in first approximation), the $orthogonal score$ follows a normalized Gaussian distribution $\mathcal {N}(0,1)$ with the usual 95% confidence interval $[-2;+2]$. The $deformation score$ would however follow a chi-squared distribution with 3 degrees of freedom ${\chi }_3^2$ (confidence interval $[0;+7.8]$).

## Data Availability

The ultrasound images analysed in the current study are not publicly available due to privacy concern. The segmented 3D surface meshes are however unidentifiable and are available, with corresponding diagnoses, on the UCL research data repository under the DOI 10.5522/04/23717376. For review purposes, the data is available at https://figshare.com/s/861ec1626bc36a562c4d and the shape model parameters at https://figshare.com/s/4bbafc08ac95523f5b86.

## Code availability

Access to the implementation of the segmentation workflow and of the statistical analysis is available on demand for review at https://gitlab.com/rsivera/segment-faces. The repository will be made publicly available before publication.
